# Bacteria−Based Synergistic Therapy in the Backdrop of Synthetic Biology

**DOI:** 10.3389/fonc.2022.845346

**Published:** 2022-04-04

**Authors:** Yawei Bao, Yong Cheng, Wei Liu, Wenguang Luo, Peijie Zhou, Dong Qian

**Affiliations:** Department of Radiation Oncology, The First Affiliated Hospital of USTC, Division of Life Sciences and Medicine, University of Science and Technology of China, Hefei, China

**Keywords:** cancer treatment, immunotherapy, bacterial therapy, chemical modification, synthetic biology

## Abstract

Although the synergistic effect of traditional therapies combined with tumor targeting or immunotherapy can significantly reduce mortality, cancer remains the leading cause of disease related death to date. Limited clinical response rate, drug resistance and off-target effects, to a large extent, impede the ceilings of clinical efficiency. To get out from the dilemmas mentioned, bacterial therapy with a history of more than 150 years regained great concern in recent years. The rise of biological engineering and chemical modification strategies are able to optimize tumor bacterial therapy in highest measure, and meanwhile avoid its inherent drawbacks toward clinical application such as bacteriotoxic effects, weak controllability, and low security. Here, we give an overview of recent studies with regard to bacteria-mediated therapies combined with chemotherapy, radiotherapy, and immunotherapy. And more than that, we review the bacterial detoxification and targeting strategies *via* biological reprogramming or chemical modification, their applications, and clinical transformation prospects.

## Introduction

Recent investigations have shown a decline in cancer mortality (lung cancer, melanoma, and so on) with the combined application of traditional and emerging therapies. Yet, it remains the primary cause of disease-related death worldwide. According to the Big Data techniques, more than 17 million cancer deaths worldwide are predicted by 2030 (1). Traditional antitumor curative treatments such as surgery and chemoradiotherapy inevitably have side effects such as the inability to eradicate cancer cells thoroughly, nonspecific cytotoxicity, and drug/radiotherapy resistance. More importantly, the highly anticipated innovative regimens such as tumor-targeted therapy and immunotherapy face challenges, namely, off-target effects, therapeutic resistance, and insufficient clinical response rate (2–4). These studies above highlight enormous challenges in cancer treatment and illustrate the significance of finding new anticancer therapies.

Encouragingly, a large number of studies have shown that some types of bacteria can selectively migrate to the tumor hypoxic area and stimulate an antitumor immune reaction, thus presented as a promising platform for cancer treatment (5). In 1868, Coley et al. attempted to use *Streptococcus pyrogenes* to infect tumor patients. It was surprising that some patients had witnessed tumor size reduction and some of them even disappeared completely, suggesting that bacterial therapy might be a valuable option (6, 7). However, the approach of Coley was questioned for a long time due to the fatal infections (6). After long-term exploration, the researchers found that specifically gene-deleted bacterial strain possessed attenuated virulence and high safety. Additionally, they found that living attenuated bacteria possess the unmatched superiorities of active targeting and specific intratumoral colonization (8).

The aforementioned advantages were attributed to the nourishing, hypoxic, and immunosuppressive features of the tumor microenvironment (TME) (9, 10). First, obligate anaerobes (such as *Clostridium* and *Bifidobacterium*) and facultative anaerobes (such as *Salmonella*, *Pseudomonas*, and *Escherichia coli*) preferentially accumulate in the high-density nutrient areas of tumors through their own specific chemical receptors, flagellar movement, and signal transduction proteins (11). Second, an inherent immune escape mechanism exists in TME to avoid the monitoring and elimination of tumor cells. Similarly, obligate or facultative anaerobic bacteria can survive without being cleared by innate immune cells such as macrophages and neutrophils or adaptive immune response (12). In addition, the deformed and damaged vascular network of TME can also promote the intratumoral infiltration and intrusion of anaerobic bacteria (13). Interestingly, a recent study has verified the aforementioned elucidation. The authors detected 1,526 human tumors and their adjacent normal tissues, namely, lung, breast, pancreas, ovary, and brain. Bacteria were found to exist intracellularly in each tumor type, with unique populations in each kind of cancer. Moreover, the bacteria in the tumor are mainly intracellular and present in both cancer cells and immune cells, indicating that they might be important components of TME (14).

In addition to the above advantage of specifically intratumoral colonization, some genetically attenuated bacterial strains are able to secrete cytotoxic proteins and stimulate potent immune reaction to kill tumor cells effectively (15). However, the failed attempts of VNP20009 in phase 1 trial indicated that combination therapy is urgently needed to make the enhancement of tumor targeting and clinical response rate possible (16). For instance, bacteria such as *Listeria monocytogenes*, *Clostridium tetani*, and *Lactobacillus acidophilus* have been applied as immunostimulants and inhibitors of tumor growth when combined with chemoradiotherapy or immunotherapy (17). More importantly, the advancements in genetic engineering combined with chemical synthesis have made bacteria-mediated tumor-targeting therapy a prospective anticancer treatment strategy to reduce systemic toxicity and improve targeting efficiency.

**Figure 1:**
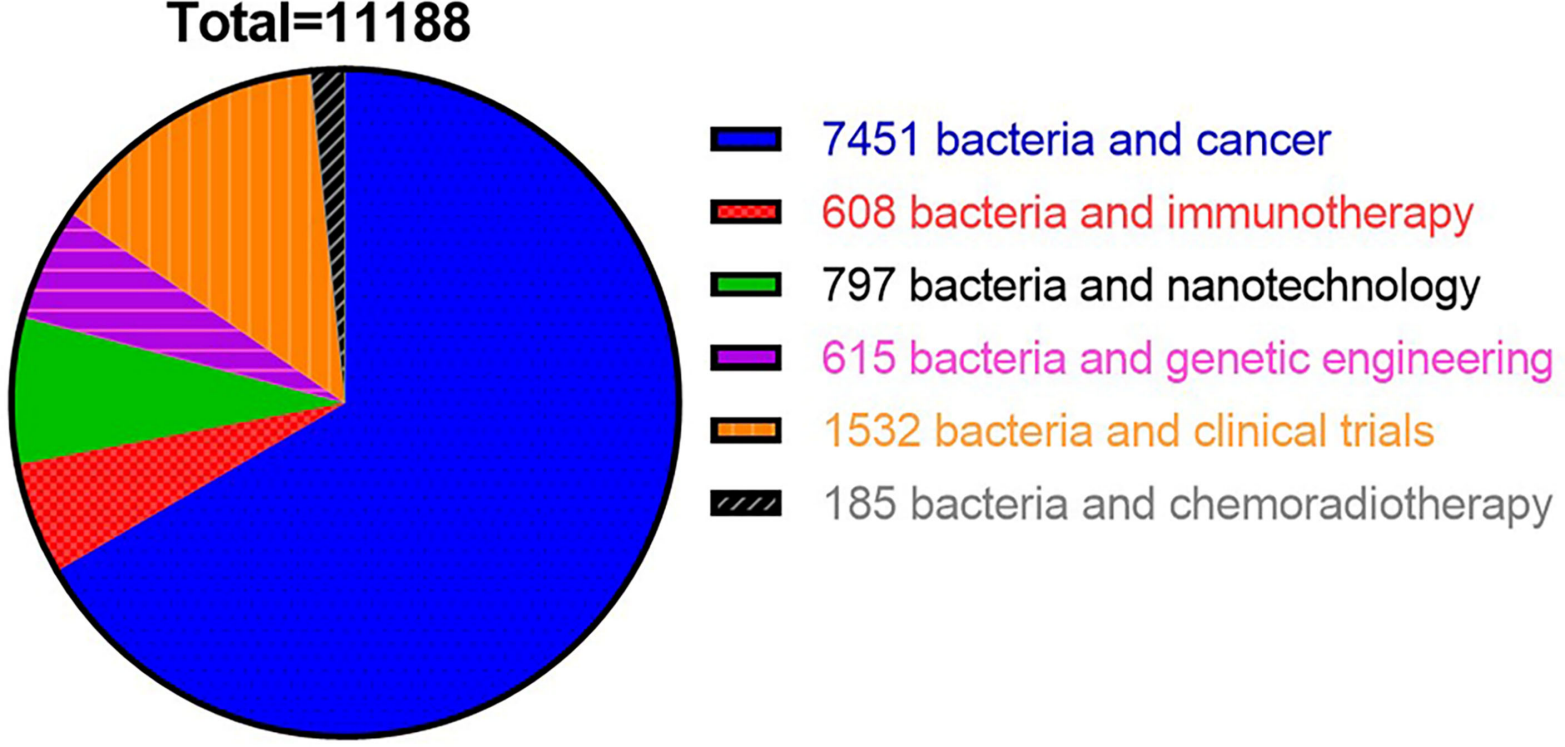
Statistical chart showed overall number of studies published in PubMed from 2012 to 2022 using different keywords.

In recent years, a mounting number of research publications on applications of bacteria−based synergistic therapy has been published. To summarize the recent progress of bacteria-based cancer therapy, we searched various keywords *via* search engine PubMed with different keywords in the past 12 years (2010–2022) and found 11,188 related papers. Among them, 7,451 studies were related to the keywords “bacteria and cancer”, followed by 1,532 studies on “bacteria and clinical trials”, 797 studies related to “bacteria and nanotechnology”, 615 documents on “bacteria and genetic engineering”, and 608 studies related to “bacteria and immunotherapy”. By comparison, the research on the combination of bacteria with chemotherapy, radiotherapy or immunotherapy is still limited (**Figure 1**). In this review, firstly, we discussed the tumor targeting properties of bacteria and the potential mechanism in the introduction. Secondly, we reviewed the bacteria-based combination therapy with chemotherapy, radiotherapy, and immunotherapy respectively. Thirdly, we reviewed the bacteria-mediated chemical modification and biological engineering. Lastly, we summarized clinical applications of bacteria-based cancer vaccines and its challenges in the future.

## Bacteria-Mediated Combined Cancer Treatment

In general, the roles of bacteria in cancer initiation and progression are a double-edged sword. On the one hand, some pathogenic bacteria can induce chronic inflammation and promote tumor development (18). *Helicobacter pylori*, as one prime example, could lead to gastric tumorigenesis through persistent inflammatory stimulation, increased epithelial cell proliferation, and deregulated signaling transduction pathways crucial for cancer maintenance (19, 20). On the other hand, some bacteria have shown great potential in treating various tumors. Bacteria can express and secrete a large number of metabolites with different biological activities that can be widely used in clinic, such as actinomycin D, doxorubicin, bleomycin, and mitomycin (21–24). Besides, a variety of enzymes, namely, L-asparaginase and arginine dehydrogenase, produced by bacteria have displayed definite anticancer efficacy (25, 26). The bacterial components and secretions are natural apoptosis inducers and immune agonists especially when employed in combination therapy (27) **(** **Table 1** **)**.

**Table 1:** Summaries of studies on bacteria with chemotherapy and radiation therapy.

Type of bacteria	Methods	Application	Outcome	References
selenium-enriched *Bifidobacterium longum*	Intraperitoneal injection	Chemotherapy	Prevention of infection in small intestinal mucositis	(28)
*Bifidobacterium longum* DD98	Preventive medication	Chemotherapy	Alleviation of intestinal and hepatic toxicities	(29)
*Salmonella typhimurium* A1-R	Targeted infection tumor	Chemotherapy	Quiescent G0/G1cancer cells to cycle to S/G2/M and chemosensitive	(30)
*Lactobacillus*	Probiotic capsules	Chemotherapy	The vaginal microbiome changes in a normal direction	(31)
*Lactobacillus kefiri* LKF01	Oral administration	Chemotherapy	Effective in preventing severe diarrhoea	(32)
*Bifidobacterium infantis*	Mixture of specific monoclonal antibody and radiation	Radiation therapy	Prevention of tumor growth and prolonged survival	(33)
*Lactobacillus acidophilus* LA-5 plus *Bifidobacterium animalis* subsp*.lactis* BB-12	Oral administration	Radiation therapy	Prevention of incidence and severity of radiation-induced diarrhoea	(34)
*Salmonella typhimurium*	Infected murine melanoma cells exposed to 8 Gy of γ-radiation	Radiation therapy	H2AX phosphorylation and apoptosis in melanoma	(35)
*Salmonella Typhimurium*	Modified miRNA expression vector encoding	Radiation therapy	Improved efficacy of radiotherapy	(36)
Heat-killed *Salmonella Typhimurium*	Intraperitoneal injection	Radiation therapy	Alleviation of radiation-induced lung injury	(37)

### Combined Bacteria-Mediated Chemotherapy

Chemotherapy, as a classical systemic treatment, is relatively effective against some types of cancer such as malignant lymphoma, childhood acute leukemia, and chorionepithelioma. Despite all these, it also has its dark side: digestive tract reaction, arrest of bone marrow, immunosuppression, and insufficient tumor targeting, particularly in acidic and hypoxic areas (38, 39). The hypoxic area of the tumor center is usually chemotherapy resistant; from this respect, anaerobes targeting the anoxic area can cooperate with chemotherapy and make up for the shortcomings perfectly (40). For example, *Salmonella*-laden temperature-sensitive liposomes (thermobots) and high-intensity focused ultrasound along with tumor heating (~40–42°C) were used to observe macrophage-related immune alterations and whether they could work in coordination with enhanced colonic chemotherapy: TB1: passively incubated TBS (mean fluorescence intensity (MFI): 8.16 ± 0.014); TB2: TBS with biotin–streptavidin (MFI: 21 ± 0.14). The activity of doxorubicin (Dox)-loaded bacteria and untreated control bacteria was 70–75% compared with *Salmonella.* The results showed that the efficacy of TB1 and TB2 was relatively higher than that in the control group at body temperature, and TB2 showed a higher killing rate than TB1 when heated (~80% vs 60%). In addition, the expression of TNF-α, IL-1β, and IL-10 in each group was significantly higher than that in the untreated control group (316 ± 53 ng/ml vs 58.3 ± 1.15 ng/ml, 84.7 ± 3.93 ng/ml, and 110.48 ± 7.82 ng/ml, respectively). In another study, the acid-unstable conjugate of maleic anhydride (ECN-Ca-Dox) was used to couple Dox with *E. coli Nissle* 1917 (ECN) to accumulate bacteria and release antibiotics. The accumulation of DOX in the tumor was 12.9 and 6.4%, respectively, 3 h and 3 days after the intravenous injection of ECN-Ca-Dox, which was much higher than that in the control group. As expected, the percentage of cell proliferation in the ECN-Ca-Dox group was significantly lower than that in the ECN-sa-Dox group (15.1 ± 1.2% vs 40.6 ± 4.3%); the rate of apoptotic cells in the ECN-Ca-Dox group increased correspondingly (41). The study next verified whether TAPET-CD (an attenuated strain of *Salmonella typhimurium* expressing *E. coli* cytosine deaminase) could convert nontoxic 5-fluorocytosine (5-FC) into active anticancer drug 5-fluorouracil (5-FU). The inhibitory effect of TAPET-CD combined with 5-FC on colon tumors after subcutaneous transplantation was evaluated. High levels of 5-FU were detected in tumors of mice treated with combined therapy, but not in normal tissues. The combined treatment had a higher inhibition of tumor growth than TAPET-CD alone (88–96% vs 38–79%). After receiving a single injection of TAPET-CD, the tumor growth was remarkably inhibited (79% on the 40th day), and the TAPET-CD/5-FC group had a notable antitumor effect (88% on the 47th day) (42).

Besides enhancing tumor killing, some probiotics can also reduce the side effects caused by chemotherapy. Chemotherapy-related intestinal catarrh of colorectal cancer is often caused by 5-FU. However, patients treated with *Lactobacillus* had less diarrhea. Also, no toxic events related to lactic acid bacteria were detected, indicating that the supplementation of *Lactobacillus rhamnosus* could enhance gastrointestinal tolerance and reduce the frequency of severe diarrhea and abdominal discomfort associated with 5-FU chemotherapy (43). One thing to note, however, is that lactic acid bacteria can also cause local infections. In rare cases, probiotics may cause systemic infections through bacteremia, especially in patients with compromised immunity or Crohn disease (44). Therefore, the safety of combined bacteria-mediated chemotherapy *in vivo* is a matter to be considered.

### Combined Bacteria-Mediated Radiotherapy

More than 60% of tumor patients need radiotherapy, yet the radiation resistance of some tumor types and decreased radiotherapy sensitivity of intratumor hypoxic areas largely account for the failure of radiotherapy (45–47). Engineered *E. coli* (5 × 10^7^ colony-forming unit (CFU)) cooperated with different doses of radiation (0, 8, 15, and 21 Gy). The combination of bacteria and 21-Gy radiation significantly reduced the tumor, completely eradicated the CT26 tumor, and dramatically inhibited tumor metastasis (48). In a recent study, Shiao et al. found that fungi and bacteria from the intestinal system in breast cancer and melanoma mouse models exhibited disparate roles. Although fungi depletion by antibiotics boosted responsiveness to radiotherapy, the bacteria exhaustion greatly accelerated tumor growth. Mechanistically, elevated Dectin-1 (intrinsic receptor of fungi infection) expression in tumor cells negatively correlated with the survival of patients with breast cancer after radiation therapy (49).

Probiotics can also exhibit the protective effect of radiotherapy. A new probiotic mixture (Microflorana-F) was tested in a male Wistar rat model of acute radiation-induced enteritis to examine the effect of supplementation of lactic acid bacteria on radiotherapy-related diarrhea in colorectal cancer. After feeding standard food and active/inactive probiotics (the same probiotics but heat inactivated) for 14 days, the changes in endotoxemia and bacterial translocation were observed. Early death (1 week) mainly occurred in rats fed standard food or inactivated probiotics. The level of endotoxin in the irradiated rats fed with standard food and inactivated probiotics increased remarkably, but the aforementioned indexes were notably improved after the addition of an active probiotic mixture (*P <*0.05). In the culture of blood, portal vein, and bile samples, active probiotics alone markedly reduced the bacterial contamination in all samples (compared with inactivated probiotics and standard feed samples, *P <*0.01) (50). In addition, probiotic *E. coli Nissle* 1917 bacteria with catalase secretion were used to relieve hypoxia of the tumor center and boost the sensitivity of radiotherapy. This bacterial strain could promote O_2_ generation and subsequent reactive oxygen species production after X-ray irradiation, and as expected, notably suppress tumor growth (51).

### Bacteria-Mediated Immunoregulation and TME Amelioration

The immunosuppressive properties of TME contribute to the immune escape of tumor cells and clinical efficacy attenuation of immunotherapy. Nevertheless, the bacteria colonized in tumor hypoxic areas are expected to remold TME and improve immune response (52). Once infected by bacteria, a large number of innate immune cells gather in the TME to kill tumor cells directly or secrete pro-inflammatory cytokines. For example, *S, typhimurium* ΔppGpp strain could activate Toll-like receptor (TLR)4 and TLR5 pathways, resulting in the massive infiltration of macrophages and neutrophils in TME and transformation of M2-like macrophages that promoted tumor progression into M1-like macrophages that inhibited tumor development (53, 54). In addition, when bacteria infected tumor cells, the release of ATP and the secretion of inflammatory bodies could trigger an inflammatory storm, which, together with cytokines or chemokines, such as IL-1β and IL-18, and pore-forming protein gasdermin D could promote tumor regression (55). Besides, Chandra et al. found that *L. monocytogenes* promoted the targeting effect of immune cells after infecting tumor cells, increased the production of IL-12 through MDSC subsets, and enhanced the cytotoxic effect of T and NK cells (56). In addition, as an indispensable part of innate immunity, TLRs and various pathogen-associated molecular patterns could be stimulated by the signals of Gram-negative bacteria, namely, lipopolysaccharide (TLR4), flagellin (TLR5), and unmethylated CpGDNA (TLR9) (57).

Apart from the innate immune system, the adaptive immune response also plays a pivotal role in bacteria-mediated antitumor therapy. Once anaerobes such as *Salmonella* entered into the tumor region, B lymphocytes and CD8^+^ T cells infiltrated and the number of regulatory T cells (Tregs) decreased to stimulate a strong tumor-killing reaction (58). Meanwhile, the anticancer activity is also exerted *via* increased expression of immunostimulatory factors (such as IL-1β, TNF-α, and IFN-γ) and inhibited immunosuppressive factors, namely, arginase-1 (Arg-1), IL-4, transforming growth factor-β (TGF-β), and vascular endothelial growth factor (VEGF) (59). Similarly, *Clostridium* infection recruits granulocytes and cytotoxic lymphocytes to TME and induces various cytokines and chemokines, thus leading to the activation of functional T cells and tumor regression (60, 61).

Tumor growth requires a special blood supply to support the oxygen and metabolic demands. Bacteria can not only eliminate cancer cells directly but also inhibit neovascularization and destroy blood vessels in the tumor tissues. Saccheri et al. found that *Salmonella* infection increased the expression of Cx43, while inhibited hypoxia-inducible factor 1α and VEGF to reduce angiogenesis in a melanoma model (62). Moreover, the upregulation of TNF-α after *Salmonella* infection promotes the permeability of blood vessels of tumor regions and leads to vascular bleeding, which subsequently contributes to the infiltration of cytotoxic immune cells (63). Therefore, apart from competing with tumor cells for nutrients and activating apoptosis or autophagy signaling pathways, bacteria can also stimulate immune responses and improve suppressive TME (64, 65).

## Chemical Modification and Biological Engineering of Bacterial Strains

Besides the synergistic reaction with the aforementioned treatments, bacteria have gained attention because of the advantage of intratumor colonization (66). Although facultative or obligate anaerobic bacteria have shown prime tumor colonization ability and are considered to be natural tumor-targeting carriers, the tumor-targeting ability and therapeutic safety of bacteria rarely go hand in hand (9, 67, 68). The main reason is that although obligate anaerobic bacteria are relatively safe and can successfully target tumors, they do not directly dissolve the tumor. In contrast, facultative anaerobic bacteria present excessive natural toxicity but may bring about obvious systemic toxicity (20). Therefore, biological engineering and chemical modification technologies are urgently needed for original bacterial strains to enhance tumor-targeting ability and acquire tolerable toxicity during systemic administration.

### Chemical Modification of Bacteria

The surface of the bacterial cell wall is electronegative. Thus, positively charged nanoparticles can be self-assembled to the surface of bacterial strains such as *Salmonella* through electrostatic interaction. Hu et al. designed a cationic nanoparticle-coated bacterial carrier assembled with a cationic polymer and plasmid DNA to synthesize nanoparticle-coated attenuated bacteria for an oral DNA vaccine in tumor immunotherapy *in vivo*. The plasmid encoding vascular *VEGFR2* gene and antigen gene could induce antigen-activated T lymphocytes and cytokines, inhibiting tumor angiogenesis and growth (69). Besides, *Bifidobacterium* (BF), a Gram-positive bacteria with a large amount of protein in the cell wall, is also negatively charged on the surface. BF was combined with cationic phase-change nanoparticles (CPNs) by electrostatic adsorption. During high intensity focused ultrasound irradiation on a tumor, BF-CPN particles could increase the energy deposition after liquid–gas phase transition. Also, the upconversion nanorods (CS-UCNR) of the core–shell structure were coated with protonated oleic acid to make its surface positively charged. *Via* electrostatic interaction and anaerobic *Bifidobacterium* UCC 2003, the imaging agent CS-UCNR was loaded and gathered on the tumor site through the anaerobic targeting of bacteria. The combination of anaerobes and functional NPs improved the treatment of tumor hypoxia and provided a novel approach for specific diagnosis and treatment (70).

Reforming bacteria by chemical bonding is another strategy due to high levels of endogenous amino groups on the cell surface. For instance, the nano photosensitizer (indocyanine green nanoparticles, INPs) is covalently bound to the surface of transgenic attenuated *S. typhimurium* strain YB1 through an amide bond. The functional INPs with the reactive carboxylic acid group (-COOH) and the amino group (-NH2) on the bacterial surface could be directly covalently linked to form a biological hybrid micro-swimmer (YB1-INP). The scanning electron microscope images showed that more than 60% INPs were attached to the surface of YB1. YB1-INP migration could be induced by the destruction of tumor tissue and the production of bacterial nutrients after photothermal treatment. The bioaccumulation of YB1-INPs was 14 times higher than that without photothermal intervention. YB1-INPs showed the characteristics of specific intratumor targeting, good photothermal conversion, and efficient fluorescence imaging and could eliminate large solid tumors without recurrence (71). In another study, poly(lactic acid-glycolic acid) copolymer PLGA nanoparticles loaded with low-boiling point perfluorohexane were integrated with anaerobic *Bifidobacterium longum* through amide bonds. The anaerobic targeted bacteria could infiltrate into the tumor deeply, increase energy deposition by affecting the acoustic environment of TME, and change the acoustic features of tumor tissue. This strain could destroy tumor cells with liquid–gas phase transition during irradiation. Thus, the addition of the bacterial anaerobic enhanced the tumor-targeting performance and retention time of administration (72).

Vesicles of cell membranes have received immense attention as delivery vectors in recent years. Nanoscale proteolipid vesicles have unmatched superiorities in drug delivery applications, namely, controllable dimensions, flexible assembly, and tractable surface modification (73). For example, bacteria-secreted outer membrane vesicles (OMVs) of attenuated Gram-negative bacteria *Klebsiella pneumoniae* along with adriamycin were prepared simultaneously and then transported to NSCLCA549 cells. Dox-OMV showed distinct tumor growth inhibition ability, good tolerance, and better pharmacokinetics. The pathogenic characteristics of OMVs containing bacterial antigens enabled macrophages to recruit in the tumor microenvironment and activate immune response (74). Further, the bacterial secretions could also be combined with nanomaterials to enhance antitumor efficacy (75). In addition, the nano-bionic pathogens were prepared by encapsulating cisplatin nanoparticles on the surface of chemotherapeutic drug cisplatin. The biomimetic nanoparticles encapsulated by OMVs were injected into tumor-bearing mice after photothermal therapy and showed a superior tumor clearance effect together with photothermal therapy (76).

### Biological Engineering of Bacteria

Bacteria have the unique ability to manipulate genes, and flagella on bacteria that can penetrate tissues make them a desirable platform to be reprogramed. Various bacteria are preferentially clustered in tumors, such as *E. coli Salmonella Bifidobacterium*, and *Clostridium* (57, 77, 78). However, unattenuated live bacteria bring about safety risks and even death due to bacterial toxins when systemically administered. To be on the safe side, the virulence-related genes must be modified *via* transposon, gene site-directed mutation, and so forth (79, 80) (**Table 2**). A new type of tumstatin drug (Tum) delivery system was established by engineering *Bifidobacterium*. The inhibitory effect of Tum transgenic *B. longum* (BL) on tumors in mice was measured. The weight, growth, and percentage of vascular endothelial cells of the transplanted tumor were also observed. After 39 days of oral (OR) administration or injection into tumors (INT) and into vena caudalis (INV), the inhibition rate in the INV-BL-Tum, INT-BL-Tum, and OR-BL-Tum groups on the transplanted tumor was 64.63, 75.21, and 38.56%, respectively. The apoptosis of tumor cells and vascular endothelial cells in the INT-Tum treatment and INV-Tum treatment groups was dramatically higher than that in the control group (*P <*0.05). All these findings confirmed the tumor-inhibitory effect of the engineered bacteria (87).

**Table 2:** Studies on engineered bacteria.

Types of bacteria	Methods	Results	Reference no.
*Escherichia coli* Nissle 1917	Encoding amino acids 45–132 of tumstatin was subcloned into inducible expression vectors and solubly expressed in *Escherichia coli* BL21	Effectively restrain mice bearing B16 melanoma tumor.	(81)
*Escherichia coli* Nissle 1917	Bearing azurin-expressing plasmids using rabbit anti-azurin polyclonal antibody.	B16 melanoma and orthotopic 4T1 breast tumor growth were restrained Pulmonary metastasis was prevented	(82)
*Escherichia coli* DH5α-lux/βG	Transforming with pRSETB-lux/βG and plasmid extraction was carried out by miniprep method	Targeted homing and proliferation in TME Tumor growth was inhibited.	(83)
*Salmonella enterica* serovar *Typhimurium*	Genetically engineered SalpIL2 was constructed by inserting the human IL-2 gene intovX^4550^ downstream	The safety of an orally in canine osteosarcoma were confirmed	(84)
*Salmonella enterica*	Modified attenuate *Salmonella enterica* released a recombinant fluorescent biomarker	Fluoromarker transport through tumor tissue Previously undetectable microscopic tumors were identified.	(85)
*Listeria monocytogenes*	Expressing mesothelin (CRS-207) with chemotherapy	Anti-tumor immune responses increased Susceptibility of neoplastic cells to immune-mediated killing enhanced.	(86)

VNP20009 is another attenuated *Salmonella* strain. The photothermal agent polydopamine (PDA) was transported to the anoxic and necrotic areas of the tumor with the tumor-targeting ability of VNP20009 to improve the antitumor effect on malignant melanoma. When the concentration of dopamine was 1,000 μg/ml, the temperature of PDA-VNP suspension increased by 23.0°C after irradiation for 300 s. However, under the same conditions, the temperature of deionized water was raised only by 8.2°C. B16F10 cells were irradiated with PDA-VNP prepared using dopamine, and 80.7% of the cells were killed. The number of bacteria in the tumor injected with PDA-VNP prominently exceeded that in other organs. The results displayed that the PDA coated on the surface of VNP20009 did not affect the targeting and colonization ability of bacteria to tumor after photothermal therapy, and the combination therapy was conducive to tumor inhibition (88). Further, Chowdhury et al. recently designed one nonpathogenic *E. coli* strain with nanobody anti-CD47 expression controllable. CD47 is a kind of “Don’t eat me” signal mainly expressed on tumor macrophages. This platform effectively activated the infiltration of cytotoxic T lymphocytes, promoted faster tumor regression, inhibited distant metastasis, and delayed the survival time of mice in the experimental group (10).

### Advantage and Disadvantages of Bacteria for Tumor-Specific Targeting and Drug Delivery

Preliminary clinical trials of bacterial cancer treatment have not been as successful as expected for several reasons. One possible reason is due to the pathogenicity of bacteria. For example, in a retrospective analysis of intravesical BCG therapy in 258 patients, complications included acute urinary retention, hematuria, and urinary tract infection (1.2% vs 2.7% vs 5.4% respectively). In addition, age is another major risk, with a higher risk of complications over the age of 80 at diagnosis (19.0% vs 7.5%, p = 0.01). Timely intervention should be performed when complications arise, and the risks and benefits of resuming intravesical BCG immunotherapy should be carefully assessed (89). In addition, in a multicenter, phase III, open and randomized controlled trial of *Lactobacillus brevis* CD2(LBCD2) for the prevention of oral mucositis in patients with head and neck tumors, a total of 68 patients were randomly divided into the intervention group (LBCD2 lozenges) and the control group (sodium bicarbonate mouthwash). Intervention and control measures were discontinued when grade 3 or 4 oropharyngeal mucositis was present during radiotherapy. The results showed that there was no significant difference between the intervention group and the control group (40.6% vs 41.6% respectively, P = 0.974), and the intervention group was similar to the control group in terms of quality of life, pain and dysphagia. However, the risk of enteral nutritional requirements was significantly reduced in the control group (OR = 0.341, 95% CI = 0.127–0.917, p <0.917). This result is related to different RT techniques, preventive measures, bacterial species, research subjects, mucositis score and others, which need to be further studied (90).

In addition to the inherent pathogenicity, another reason dampening bacteria-based therapy is that in animal models, the toxicity is minimal due to the strong targeted colonization of bacteria and the small number of bacteria required. However, when translated into human trials, the number of bacteria used for treatment and the space of necrosis within the tumor need to be calculated and evaluated more accurately (91). Besides, the comprehensive roles of bacteria therapy are quite complicated in different tumor context. Recent studies have found that microbial regions promote the molecular pathogenic mechanism of cancer initiation and development. in the chemotherapy resistance of colorectal cancer patients, *Fusobacterium* was abundant in recurrent colorectal cancer tissues after therapy (92). In addition, castrated-resistant prostate cancer mice and patients have rich intestinal microflora, which makes androgen precursors converted into active androgens and participate in tumor drug resistance (93).

The characteristics of hypoxia in tumor tissue, especially in the central area, make the tumor resistant to radiotherapy, which ultimately leads to poor therapeutic effect (94). Gold nanoparticles (GNPs) with the characteristics of evading immune system and targeting tumor become a suitable radiosensitizer for radiotherapy, but its delivery effect in the anoxic area of the tumor center is not good, so a tool that can targeted transport GNPs is needed to make up for the effect of radiotherapy in the hypoxic area. Previous studies have shown that bacteria can selectively colonize in anoxic sites and active in these areas. *S. typhimurium* as a highly active delivery agent has been reported by many studies on hypoxic regions of tumors. Amirhosein et al. (95) used live attenuated *Salmonella* Typhi Ty21a with folic acid functionalized GNPs (FA-GNPs) to obtain the Golden Bacteria, then injected FA-GNPs into the tail vein of CT-26 tumor-bearing mice, and calculated the ratio of periphery regions of tumors in comparison with central regions of tumors. The result of FA-GNPs injection group and Golden Bacteria group was 1.95 ± 0.13 vs 0.61 ± 0.10. This observation demonstrates that even if GNPs modified with folic acid targeted cancer cells, it still reached the periphery of the tumor rather than the center of the tumor. The main reason is that the vascular system around the tumor is different from that in the central area, the surrounding blood vessels are more mature and dense, intelligent targeting is still unable to use systemic circulation for effective treatment in hypoxia areas. However, the flagellum movement of anaerobic bacteria can be a means of transport in anoxic zone to change this dilemma. The bacteria in this study are safe as an active carrier in tumor-bearing mice. and there was a significant advantage in transporting GNPs to the central hypoxic area of the tumor.

## Clinical Application of Bacteria-Based Cancer Vaccine

Bacteria-based cancer vaccine is a crucial application of bacteria toward clinical transformation. Cancer vaccines mainly include four components: vectors, various formulations, cancer adjuvants, and specific antigens. Among these, cancer-specific antigens may be the most concerning section determining the effectiveness and specificity of tumor vaccines (96–98). Through chemical modification and biological engineering as mentioned earlier, bacteria present considerable foreground of clinical application. When the virulence is attenuated, bacteria display great potential of exerting an antitumor effect. The inherent features of bacteria make them effective immunostimulants (99, 100).

Bacillus Calmette–Guérin (BCG), the only recognized and licensed live bacteria for cancer treatment, was an attenuated strain of *Mycobacterium bovis* and was successfully applied by Morales in 1976 to treat superficial bladder cancer (BC) (101, 102). Nowadays, BCG has become a significant choice for treating high-risk superficial BC in most countries (103). A randomized trial compared the efficacy of intravesical maintenance therapy with BCG and radical cystectomy (RC) in treating high-grade non-muscular invasive bladder cancer (NMIBC). Of the 23 patients treated with BCG, 4 developed NMIBC after induction, 3 developed NMIBC after 4 months, and 2 had metastatic cancer. The 20 patients who underwent RC treatment, 5 had no tumor, 13 had highly malignant NMIBC, and 2 were detected with muscle infiltration. The adverse reactions in both groups were mild [15/23 (65.2%) BCG vs 13/20 (65.0%) RC] with similar quality of life. The results showed that a considerable number of patients were suitable for bladder preservation and could contribute to health and quality of life (104).

Recently, Alejandrina et al. attempted to verify the treatment potential of *S. typhimurium* vaccine strain CVD915 with the liver metastasis model of breast cancer and lymphoma models. After 21 days, tumor infiltration was observed in both tumor models. In addition, the expression of tumor-suppressive IL-10 levels and the number of neutrophils and regulatory T cells decreased. In the lymphoma model, about 10% of the mice witnessed the elimination of tumor growth. The tumor-specific Th1 reaction was triggered in the CVD915 group, followed by an increase in the number of CD4^+^ T cells and dendritic cells. Meanwhile, the number of tumor nodules in the liver decreased by 50%, and the tumor volume decreased by 45% (105).

The use of attenuated *Listeria*, as a bacterial vector for cancer vaccines, has been widely reported in preclinical trials (106, 107). Taking cervical cancer as an example, the cancer-specific antigen human papillomavirus type 16 E7 (HPV-16 E7)was fused with the attenuated sequence Listeriolysin O (LLO) of hemolysin protein to establish therapeutic cervical cancer vaccines. The vaccines based on attenuated *Listeria* delayed tumor progression *in situ* (108) and provided long-term immunity for patients with early-stage cervical cancer (109). This vaccine could induce robust immunity reaction and proliferation of cytotoxic T cells. It had unique advantages such as genomic nonintegration and could be exhausted easily *via* antibiotics to avoid serious side effects (110–112). Therefore, taking advantage of live bacteria as biological carriers to deliver recombinant tumor-specific antigens is an effective approach to develop cancer vaccines.

Andreas et al. studied an oral DNA vaccine VXM01 that induced an immune response against vascular endothelial growth factor receptor 2 (VEGFR2) in patients with stage IV and locally advanced pancreatic cancer (113). The vaccine used a licensed live and attenuated *S. typhimurium* strain Ty21a as vectors. Subsequently, 18 patients with advanced pancreatic cancer receiving VXM01 and 8 patients receiving placebo (isotonic sodium chloride). The oral vaccine was given four times on the first, third, fifth, and seventh days, while the fortified vaccine was given six times per month after the last vaccination. The results showed that 75% (3/4) of the patients in the high-dose group and 66.7% (8/12) in the low-dose group had a 1.8-fold increase in T cell response compared with the placebo group. In addition, patients receiving high-dose VXM01 vaccination showed a generally strong anti-VEGFR2 response (114). Furthermore, the safety and immunogenicity of the recombinant *Lactococcus lactis* vaccine expressing the HPV type 16 E7 oncogene were evaluated in a phase I safety and immunogenicity test of healthy female volunteers (115, 116). A total of 55 qualified volunteers were divided into vaccine and placebo groups. Compared with the placebo group, a specific IgG immune response could be induced 30 days after vaccination in the 1 × 10 ^9^ CFU/ml group compared with the placebo group (5 × 10^9^ CFU/ml vs 1 × 10^10^ CFU/ml, *P* = 0.0137 vs *P* = 0.0018). This study showed that the candidate HPV16 E7 oncoprotein oral vaccine produced by *Lactobacillus* was safe and immunomodulatory (**Table 3**).

**Table 3:** Selected clinical trials investigating bacteria and cancer vaccine.

Trial number	Therapeutic agent	Population	Mode of delivery	Stage of trial	Country
NCT02302170*	*Helicobacter pylori* vaccine	Healthy children aged 6–15 years	Oral vaccination	Phase 3	China
NCT00736476*	*Helicobacter pylori* antigens-vacuolating cytotoxin A, cytotoxin-associated antigen and neutrophil-activating protein	Healthy non-pregnant adults aged 18–40 years	Intramuscular injection	Phase 1/2	Germany
NCT02371447*	Recombinant *Bacillus* Calmette–Guérin (VPM1002BC)	Patients with intermediate to high risk and recurrent NMIBC	Intravenous infusion	Phase 1/2	Switzerland
NCT02243371*	*Listeria* monocytogenes-expressing mesothelin (CRS-207)	Patients with cytologically or histologically-proven, metastatic adenocarcinoma of the pancreas	Intravenous infusion	Phase 2	USA
NCT01838200*	*Bacillus* Calmette–Guérin	Patients with unresectable stage III or stage IV melanoma	Subcutaneous injection	Phase 1	Australia

*ClinicalTrials.gov identifier.

## Challenges and Future Prospective

**Figure 2:**
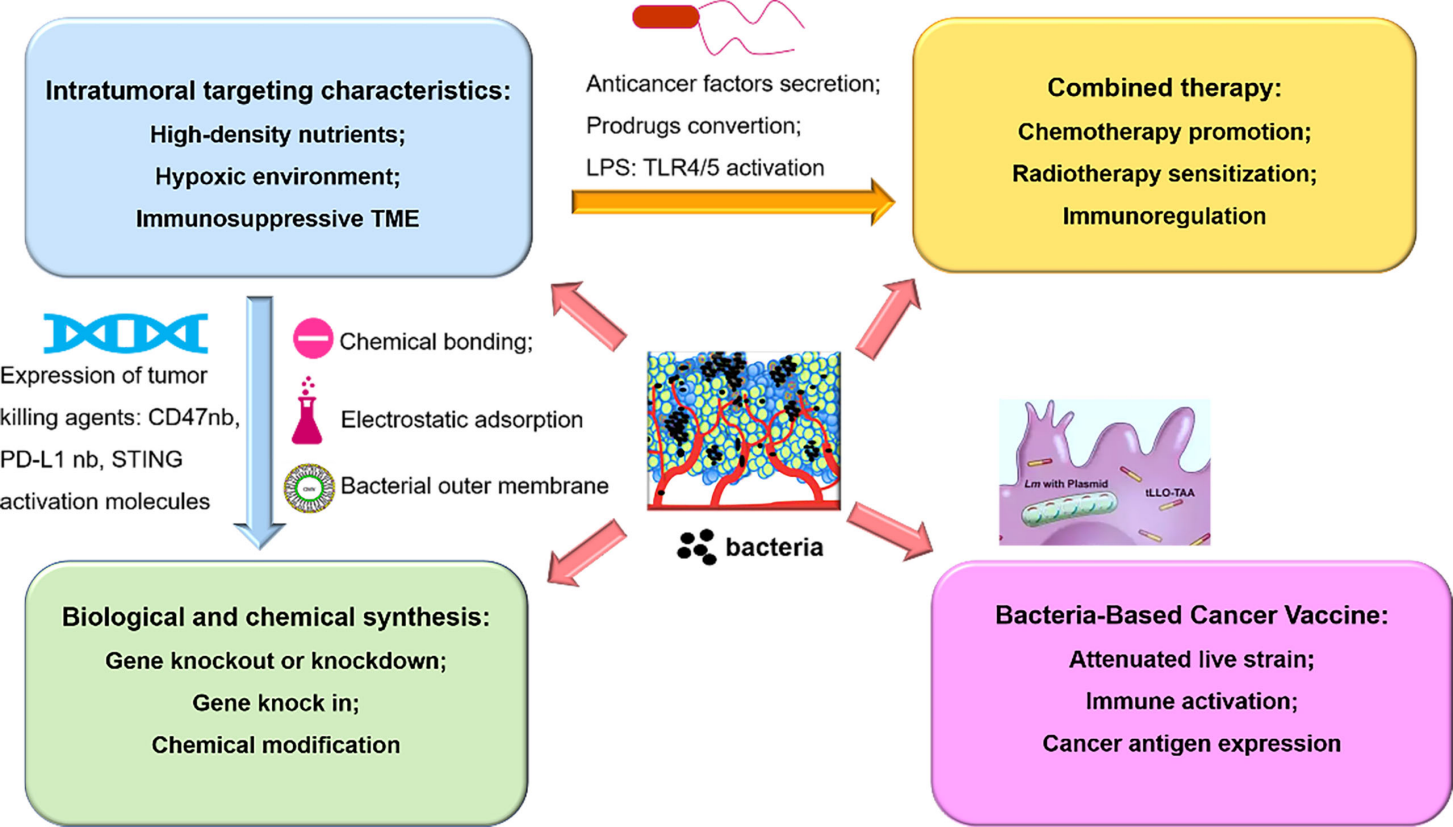
An overview of bacteria-based synergistic therapy, namely, the mechanism of tumor targeting properties, combined therapy with chemoradiotherapy or immunotherapy, chemical modification or biological engineering, and bacteria-mediated delivery of cancer vaccine.

Although bacteria have potent cytotoxicity (such as Coley’s toxins) and powerful intratumor colonization capabilities which make them desirable tumor killing agents and delivery platform, obstacles toward clinical applications has been there all along. One of the main challenges is that they may cause immune side effects due to the inherent immunogenicity. Novel biological and chemical modification can expand bacteria-mediated clinical transformation, because researchers can obtain bacteria with maximal advantages *via* knockout related genes to reduce pathogenicity while retaining functions of bacteria. In addition, timely management of effective antibiotics can also reduce the risk of severe infection. Secondly, in consideration of the dose-dependent toxicity presented in previous clinical trials, it seems difficult for bacteria to be administered multiple times. Thus, limited drug loading capacity is another challenge dampening the applications of attenuated bacteria strains. Interestingly, how to transform bacteria into intelligent “bacterial factory” or “bacterial machine” appears to be emerging research highlights (117, 118). The combined applications of robot technology and biotechnology are expected to promote bacteria to move intelligently to tumor sites and increase drug loading capacity at the same time (**Figure 2**).

## Conclusions

The unmatched advantage of tumor-targeting properties make bacteria the ideal oncolytic agent to kill tumor cells specifically and the excellent platform to deliver multifarious drugs. However, bacteria-mediated therapy alone can hardly eliminate tumor cells completely. As bacteria therapy has two sides, a large number of clinical studies are needed to balance the role of both good and evil of bacteria-based therapy when combined with chemotherapy agents, radiation or immunotherapy. So far, BCG is the only viable curative treatment approved by the FDA up to the present. Although cancer vaccines based on attenuated *Listeria* have entered clinical trials of phase III, there are still some difficulties to be addressed such as bacterial virulence, instability of expression plasmid in bacteria, drug delivery efficiency intracellular. Development of genetic engineering approaches, optimization of chemical modification process and selection of targeted reagents such as tumor specific antigen peptide are of supreme importance in the near future.

## Author Contributions

YB: Draft writing, reviewing and data processing. YC: Draft supervision and data checking. WLi: Draft reviewing and data checking. WLu: Draft reviewing and data checking. PZ: Draft writing, reviewing and funding acquisition. DQ: Draft supervision and investigation. All authors listed have made a substantial, direct, and intellectual contribution to the work and approved it for publication.

## Funding

This work was supported by the National Natural Science Foundation of China (82102953) and the Natural Science Foundation of Anhui Province (1908085QH338).

## Conflict of Interest

The authors declare that the research was conducted in the absence of any commercial or financial relationships that could be construed as a potential conflict of interest.

## Publisher’s Note

All claims expressed in this article are solely those of the authors and do not necessarily represent those of their affiliated organizations, or those of the publisher, the editors and the reviewers. Any product that may be evaluated in this article, or claim that may be made by its manufacturer, is not guaranteed or endorsed by the publisher.
